# International urogynecology consultation chapter 3 committee 2; conservative treatment of patient with pelvic organ prolapse: Pelvic floor muscle training

**DOI:** 10.1007/s00192-022-05324-0

**Published:** 2022-08-18

**Authors:** Kari Bø, Sònia Anglès-Acedo, Achla Batra, Ingeborg Hoff Brækken, Yi Ling Chan, Cristine Homsi Jorge, Jennifer Kruger, Manisha Yadav, Chantale Dumoulin

**Affiliations:** 1 Department of Sports Medicine, The Norwegian School of Sport Sciences, PO Box 4014, Ullevål stadion, 0806 Oslo, Norway; 2 Department of Obstetrics and Gynecology, Akershus University Hospital, Lørenskog, Norway; 3 Urogynaecology Unit Hospital Clínic de Barcelona, Barcelona, Spain; 4 Department of Obstetrics & Gynaecology, VMMC & Safdarjung Hospital, New Delhi, India; 5 Kolbotn Physical Institute, Nordre Follo Municipality, Nordre Follo, Norway; 6 The Pelvic Floor Centre, Division of Surgery, Akershus University Hospital, Lørenskog, Norway; 7 Department of Obstetrics and Gynaecology, Calderdale and Huddersfield NHS Foundation Trust, Huddersfield, UK; 8 Department of Health Science Ribeirão Preto Medical School, University of São Paulo, São Paulo, Brazil; 9 Auckland Bioengineering Institute, University of Auckland, Auckland, New Zealand; 10 Paropakar Maternity and women’s hospital, Thapathali, Kathmandu, Nepal; 11 School of rehabilitation, Faculty of Medicine, Université de Montréal, Montréal, Canada

**Keywords:** Long-term, Pelvic floor muscle training, Physical therapy, Pelvic organ prolapse, Postpartum, Surgery

## Abstract

**Introduction and hypothesis:**

This manuscript from Chapter 3 of the International Urogynecology Consultation (IUC) on Pelvic Organ Prolapse (POP) describes the current evidence and suggests future directions for research on the effect of pelvic floor muscle training (PFMT) in prevention and treatment of POP.

**Methods:**

An international group of four physical therapists, four urogynecologists and one midwife/basic science researcher performed a search of the literature using pre-specified search terms on randomized controlled trials (RCTs) in Ovid Medline, EMBASE, CINAHL, Cochrane, PEDro and Scopus databases for publications between 1996 and 2021. Full publications or expanded abstracts in English or in other languages with abstracts in English were included. The PEDro rating scale (0–10) was used to evaluate study quality. Included RCTs were reviewed to summarize the evidence in six key sections: (1) evidence for PFMT in prevention of POP in the general female population; (2) evidence for early intervention of PFMT in the peripartum period for prevention and treatment of POP; (3) evidence for PFMT in treatment of POP in the general female population; (4) evidence for perioperative PFMT; (5) evidence for PFMT on associated conditions in women with POP; (6) evidence for the long-term effect of PFMT on POP. Full publications in English or in other languages with abstracts in English and expanded abstracts presented at international condition specific societies were included. Internal validity was examined by the PEDro rating scale (0–10).

**Results:**

After exclusion of duplicates and irrelevant trials, we classified and included 2 preventive trials, 4 trials in the post-partum period, 11 treatment trials of PFMT for POP in the general female population in comparison with no treatment or lifestyle interventions, 10 on PFMT as an adjunct treatment to POP surgery and 9 long-term treatment trials. Only three treatment studies compared PFMT with the use of a pessary. The RCTs scored between 4 and 8 on the PEDro scale. No primary prevention studies were found, and there is sparse and inconsistent evidence for early intervention in the postpartum period. There is good evidence/recommendations from 11 RCTs that PFMT is effective in reducing POP symptoms and/or improving POP stage (by one stage) in women with POP-Q stage I, II and III in the general female population, but no evidence from 9/10 RCTs that adding PFMT pre- and post -surgery for POP is effective. There are few long-term follow-up studies, and results are inconsistent. There are no serious adverse effects or complications reported related to PFMT.

**Conclusions:**

There are few studies on prevention and in the postpartum period, and the effect is inconclusive. There is high-level evidence from 11 RCTs to recommend PFMT as first-line treatment for POP in the general female population. PFMT pre- and post-POP surgery does not seem to have any additional effect on POP. PFMT is effective and safe but needs thorough instruction and supervision to be effective.

## Introduction

This report is part of a series of articles that are the product of the International Urogynecology Consultation (IUC) sponsored by the International Urogynecological Association (IUGA). This is a 4-year, 4-chapter project with 16 reports dedicated to reviewing and summarizing the world’s literature on pelvic organ prolapse (POP). This report is from the third year and chapter of the project, which is dedicated to reviewing the literature on non-surgical management of POP. Years 1 and 2 were dedicated to defining the epidemiology of POP and the evaluation of patients with POP. The fourth or final chapter will evaluate the surgical treatment of POP. This report focuses on reviewing the literature on conservative treatment of patient with POP employing pelvic floor muscle training.

POP is a common condition in women. The reported prevalence varies widely (1–65%) based on whether its presence is ascertained by symptoms (1–31%), pelvic examination (10–50%) or both (20–65%) [1]. POP may negatively affect quality of life, participation in physical activities, body image and sexual function [2]. Multiple factors contribute to the development of primary and recurrent POP. These factors include childbirth, constipation, strenuous work and heavy lifting, congenital connective tissue weakness, obesity, menopause, chronic increased intra-abdominal pressure (IAP) and iatrogenic causes [1, 3]. Available treatments are watchful waiting, lifestyle interventions such as avoiding constipation and straining, pelvic floor muscle training (PFMT), pessaries or surgery.

POP is caused by a combination of impairment of the pelvic floor muscles (PFM) and the connective tissue [4]. The association between a decrease in levator plate angle, increase in levator hiatus area and presence of POP has been confirmed in several studies [5–13]. Women with POP have shown to have PFM impairment [4, 6, 8–16], and damage to the PFMs is three times more likely to be found in women with POP than in those without [4]. A recent systematic review by Siahkal et al. (2021) [10] summarized 21 studies in a meta-analysis and concluded that the dimensions of the levator hiatus are greater both at rest and during Valsalva maneuver in women with POP compared to women without POP. An increased levator hiatus area, measured by ultrasound, is associated with a low vaginal resting pressure, measured with manometry [17], and major tears of the PFM after vaginal birth result in a 50% reduction in muscle strength [18]. Women with Pelvic Organ Prolapse Quantification system (POP-Q) stage II or more have 40–62% reduced PFM strength [14, 19], 53% reduced PFM endurance and lower vaginal resting pressure compared to matched controls with normal supports, defined as POP-Q stage 0 and I [19]. When measuring PFM strength with manometry one longitudinal cohort study found that for every 5-cmH_2_O increase in pressure during maximal voluntary contraction, the odds for prolapse was reduced by 13% (OR 0.87, 95% CI 0.81, 0.94) [15].

Kegel (1952) [20] postulated that “With adequate PFMT the woman learns to maintain the perineum, bladder and uterus in a higher position, the slack in the supportive muscles will be taken up, and the vagina will become longer and tighter.” Today there is evidence for two mechanisms of how PFMT may be effective in prevention and treatment of SUI [21, 22], and the same mechanisms apply for the effect of PFMT to prevent and treat POP. The two mechanisms are:

(1) Women learn to consciously contract the PFM before and during increases in intra-abdominal pressure (IAP) (also termed ‘bracing’ or ‘performing the Knack’) and continue to perform such voluntary contractions as a behavior modification to prevent descent of the pelvic floor during these episodes.

(2) Women are taught to perform regular strength training to improve ‘firmness’ and structural support of the pelvic floor over time. These two hypotheses co-exist as they both build on an anatomical and biomechanical understanding of the function of the PFM, i.e., a voluntary contraction of the PFM constricts the levator hiatus, elevates and stabilizes the pelvic floor into a higher position. This results in an immediate effect during the PFM contraction of lifting the bladder neck and stabilizing the pelvic floor [23, 24]. This may also have an effect over time. Strength training has been shown to promote hypertrophy of targeted muscles, increasing constriction of the levator hiatus, shortening the PFM and lifting of the pelvic floor and reversing some of the changes associated with POP [25].

This narrative review summarizes the current evidence for PFMT in the treatment and prevention of POP with the following research questions:

1. What is the evidence for PFMT in prevention of POP in the general female population?
2. What is the evidence for early intervention of PFMT in the peripartum period for treatment of POP?
3. What is the evidence for PFMT in treatment of POP in the general female population?
4. What is the evidence for PFMT pre- and post POP surgery?
5. What is the evidence for PFMT on associated conditions in women with POP?
6. What is the evidence for long-term effect of PFMT in treatment of POP?

## Methods

To complete an exhaustive literature search on this topic, the authors brainstormed search terms they felt most relevant to POP and selected the items listed below. This list of terms was presented at the IUGA annual scientific meeting in 2020 for input from the general membership. No additional terms were suggested as a result of that meeting. The search strategy included the following: Ovid Medline, EMBASE, CINAHL, Cochrane, PEDro and Scopus databases were searched for period January 1, 1996, to December 31, 2021, to identify studies. The keywords were combinations of “pelvic organ prolapse” or “urogenital prolapse” and “bulge” or “heaviness,” “exercise therapy,” “hypopressive exercise,” “kegel,” “long term,” “pelvic floor muscle training,” “pelvic floor exercise,” “peripartum,” “postpartum,” “pregnancy,” “prevention,” “symptomatic prolapse” and “treatment outcome”. Limitation was set on fully published randomized controlled trials (RCTs) or full publications with at least an English language abstract. The search also included the extended abstracts from International Continence Society (ICS) meetings. Finally, the authors hand-searched publications from International Consultation on Incontinence (ICI) [26], NICE (2019) [27] and the book chapter on PFMT for POP from Bø and Frawley (2015) [28]. Reference lists from studies initially selected were also searched to identify any additional relevant studies not identified by the electronic searches. RCTs using other exercise programs than PFMT and comparison between PFMT and other exercise programs for POP are excluded from this review and will be published in a separate manuscript.

The PEDro rating scale, widely used in physical therapy and rehabilitation science [29, 30], was applied to assess the risk of bias (internal validity) for the RCTs. Table 1 shows the ten criteria used in this rating scale. Criteria E (eligibility) concerns external validity (generalizability). A total PEDro score less than four is considered poor, four to five fair, six to eight good and nine to ten excellent [31]. The PEDro scale (Table 1) has been found to be a reliable and valid tool to evaluate methodological quality in clinical trials [30, 32, 33].

**Table 1:** PEDro quality scores of RCTs

E: Eligibility criteria specified	
1. Subjects randomly allocated to groups	
2. Allocation concealed	
3. Groups similar at baseline	
4. Subjects blinded	
5. Therapist administering treatment blinded	
6. Assessors blinded	
7. Measures of key outcomes obtained from > 85% of subjects	
8. Data analysed by intention to treat	
9. Statistical comparison between groups conducted	
10. Point measures and measures of variability provided	

+: criterion is clearly satisfied, -: criterion is not satisfied, ?: not clear if the criterion was satisfied. Total number is determined by counting the number of criteria that are satisfied, except “eligibility criteria specified” score is not used to generate the total score. Total scores are out of 10

Methods, definitions, and units conform to the standards jointly recommended by the International Urogynecological Association and the ICS except where specifically noted [34–36].

## Results

### Outcome measures

A plethora of different outcome measures have been used in published studies on PFMT to treat POP. Table 2 gives an overview of the different measures found in the included studies. Most of the studies reported on training adherence, using appointment attendance and self-reported daily diary entries as outcome measures. Less than half reported on adverse events/complications related to PFMT. For primary outcome measures most of the studies used validated, condition-specific, self-reported questionnaires for symptoms, and the Pelvic Organ Prolapse Quantification (POP-Q) exam results for anatomic description. Few studies assessed muscle morphology using ultrasonography. A minority of the studies focused on cost analyses or need for further POP treatment.

**Table 2:** Outcome measure used in randomized controlled trials (RCTs) of pelvic floor muscle training to prevent or treat pelvic organ prolapse. Classification according to primary/secondary measures

Primary outcome:	
• Stage of POP	
1. POPQ (Braekken 2010b, Panman 2017, Wiegersma 2014, Hagen 2014, Barber 2014, Weidner 2017, Jelovsek 2018, Nyhus 2020, Due 2016a, Due 2016b, Hagen 2009, Stüpp 2011, McClurg 2014, Kashyap 2013, Pauls 2013, Pauls 2014, Panman 2016)	
• Positions of organs (imaging, observation)	
1. Position of bladder and rectum by 3D-transperineal ultrasound (Braekken 2010b, Braekken 2010a, Nyhus 2020)	
2. Anatomic failure/surgical failure (combined outcome: POPQ and no re-treatment) (Barber 2014, Wiedner 2017, Jelovsek 2018)	
• Symptoms of POP (not bladder symptoms)	
1. Specific questions yes/no	
2. POP-SS (Hagen 2017, Hagen 2014, Hagen 2009, McClurg 2014, Kashyap 2013)	
3. Validated POP-specific questionnaire to describe frequency and bother of prolapse (Braekken 2010b, Braekken 2015)	
4. PFDI-20/*UDI-6/POPDI-6/CRADI-8* (Panman 2016, Panman 2017, Wiegersma 2014, Barber 2014, Jelovsek 2018, Duarte 2020, Due 2016a, Due 2016b, Cheung 2016, Pauls 2013, Pauls 2014)	
5. Number of days with prolapse symptoms in the previous 4 weeks (Hagen 2014)	
6. VAS 0-100 POP symptoms (Nyhus 2020, Kashyap 2013)	
7. Rating of women’s perception of health benefit (better, same, worse). (Hagen 2017, Hagen 2014, Hagen 2009)/symptoms same/better/worse (Wiegersma 2014, Cheung 2016)	
8. PGI-I (Panman 2016, Panman 2017, Weidner 2017, Jelovsek 2018, Duarte 2020, Due 2016a)	
9. VAS 0-10 improvement/deterioration (Wiegersma 2014)	
10. Bulge/lump, pelvic heaviness, backache of P-QoL (Stüpp 2011)	
11. Obstructive symptoms of POP as detailed of UDI-19 (Frawley 2010)	
• Muscle morphology by transperineal ultrasound (TPUS)	
1. Levator ani muscle trauma (Nyhus 2020)	
2. Cross-sectional area levator ani muscle by 2D transperineal ultrasound and 3D TPUS (Braekken 2010a)	
3. Levator ani muscle length at rest and during max Valsalva by 3D TPUS (Braekken 2010a)	
4. Pubovisceral muscle thickness at rest by 3D-TPUS (Braekken 2010a)	
• Quality of life	
• POP-qual (Brandt and Janse Van Vuuren, 2020)	
• Cost analyses and need of POP-treatment	
1. The use of absorbent pads (Panman 2016, Panman 2017)	
2. EuroQoL 5D-3L/SF-6-→ QUALY/ICER/ ICUR (Panman 2016, Panman 2017, Hagen 2017)	
3. Use of health services in primary and secondary care (Hagen 2014)	
4. Uptake of treatment for prolapse symptoms during the follow-up [Hagen 2017, Hagen 2014, Barber 2014, Jelovsek 2018 (combined outcome: POPQ and no re-treatment), Due 2016a, Due 2016b, Panman 2016, Panman 2017]	
Secondary outcomes:	
• Sexual symptoms/ dysfunction	
1. ICIQ-VS (Hagen 2017, Hagen 2014, Hagen 2009)	
2. PISQ12 (Hagen 2017, Panman 2016, Panman 2017, Wiegersma 2014, Hagen 2014, Weidner 2017, Duarte 2020, Due 2016a, Due 2016b, McClurg 2014, Pauls 2013, Pauls 2014)	
3. Validated POP-specific questionnaire to describe frequency and bother of sexual symptoms (Braekken 2015)	
4. Frequency of sexual intercourse, satisfaction with the frequency of intercourse, and the extent of their sexual issues, reasons for not having sex (Braekken 2015)	
5. Semi-structured interview (changes in sexual desire, orgasm frequency, intensity, ability to achieve orgasm); perception of dryness, burning or discomfort/pain; self-confidence regarding sex, and/or if there were any other changes they experienced (Braekken 2015)	
6. FSFI (Pauls 2013, Pauls 2014)	
• Bladder/urinary symptoms (pad test, questionnaire)	
1. Bladder diary (Pauls 2013, Pauls 2014, Frawley 2010)	
2. Pad test (Frawley 2010)	
3. ICIQ-IU-SF (Hagen 2017, Braekken 2010b, Hagen 2014, Hagen 2009, McClurg 2014)	
4. UDI-6 (Panman 2017, Wiegersma 2014, Barber 2014, Panman 2016, Pauls 2014, Jelovsek 2018, Due 2016a, Due 2016b, Panman 2016, Duarte 2020, Cheung 2016)	
5. UDI-19 (Frawley 2010)	
6. Validated POP-specific questionnaire to describe frequency and bother of bladder (Braekken 2010b, Braekken 2015)	
7. ISI (Barber 2014, Jelovsek 2018)	
8. Bladder symptoms of P-QoL (Stüpp 2011)	
• Bowel symptoms	
1. CRADI-8 (Panman 2017, Wiegersma 2014, Panman 2016, Pauls 2014, Barber 2014, Jelovsek 2018, Due 2016a, Due 2016b, Panman 2016, Duarte 2020, Cheung 2016)	
2. ICQ-BS (Hagen 2017, Hagen 2014, Hagen 2009, McClurg 2014)	
3. Modified Wexner score (Frawley 2010)	
4. Constipation scoring system (Frawley 2010)	
5. Validated POP-specific questionnaire to describe frequency and bother of bowel (Braekken 2010b, Braekken 2015)	
6. Bowel symptoms of P-QoL (Stüpp 2011)	
• QoL	
1. P-QoL (Stüpp 2011)	
2. SF-12 (Hagen 2017, Hagen 2014, Hagen 2009, McClurg 2014, Pauls 2014)	
3. SF-36 (Weidner 2017)	
4. PFIQ**-7**/*UIQ-7/POPIQ-7/CRAIQ-7* (Panman 2016, Panman 2017, Wiegersma 2014, Weidner 2017, Jelovsek 2018, Duarte 2020, Due 2016a, Due 2016b, Cheung 2016, Kashyap 2013, Pauls 2013, Pauls 2014)	
5. MOS-SF12/*PCS-12/MCS-12* (Panman 2016, Panman 2017, Wiegersma 2014)	
6. Quality of life: interference/bother of prolapse symptoms with everyday life, scored 0 [not at all] to 10 [a great deal]): VAS (Hagen 2014, Hagen 2009, Cheung 2016)	
7. World Health Organization Quality of Life (WHOQOL)-BREF (Pauls 2013, Pauls 2014)	
8. IIQ-7 (Frawley 2010)	
9. Assessment of Quality of Life (AQoL) score (Frawley 2010)	
• Changes of lifestyle	
1. Lifestyle changes (Hagen 2017, Hagen 2014)	
• Body image	
1. Normalized body image questionnaires (Weidner 2017)	
• Need of treatment for dyspareunia, urinary symptoms	
1. Uptake of treatment for urinary symptoms during the follow-up (Barber 2014, Jelovsek 2018)	
2. Uptake of treatment for dyspareunia during the follow-up (Weidner 2017)	
3. Desired treatment before any intervention/end treatment: patient’s preference (Cheung 2016)	
Independent variables/outcomes:	
• PFM function/impairment	
1. By manometry (Braekken 2010b, Braekken 2015, Duarte 2020, Nyhus 2020, Frawley 2010)	
2. By surface electromyography during the rest and during the maximum voluntary contraction (Resende 2012, Nyhus 2020, Stüpp 2011, Pauls 2013, Pauls 2014)	
3. By digital palpation of:	
• Maximum voluntary contraction/modified Oxford scale and muscular endurance/PERFECT (Resende 2012, Nyhus 2020, Hagen 2009, Stüpp 2011, McClurg 2014, Pauls 2013, Pauls 2014, Frawley 2010)	
• Normal/underactive/overactive/inactive (Panman 2017, Wiegersma 2014, Panman 2016)	
• Strength Brink grading system (Barber 2014, Weidner 2017)	
4. By transperineal ultrasound (TPUS)	
• Change in levator ani muscle length at rest and during max Valsalva by 3D TPUS (Braekken 2010a)	
• Change in levator hiatal dimensions from rest to maximum PFM contraction by 3D transperineal ultrasound (Braekken 2010a, Nyhus 2020)	
• Training diary	
1. Appointments attendence and self-reported daily diary (Hagen 2017; Braekken 2010b, Braekken 2010a, Braekken 2015; Hagen 2014; Barber 2014, Weidner 2017, Jelovsek 2018; Duarte 2020; Nyhus 2020; Hagen 2009; Stüpp 2011; McClurg 2014; Kashyap 2013; Frawley 2010)	
• Complications regarding PFMT treatment including bother of PFMT	
1. Complications/adverse events (Cheung 2016, Braekken 2010b, Braekken 2010a, Braekken 2015, Hagen 2014, Panman 2017, Wiegersma 2014, Hagen 2017, Panman 2016, Weidner 2017, McClurg 2014, Jelovsek 2018)	
2. Bother of PFMT by numeric rating scale 0–10 (due 2016a, due 2016b)	

### Evidence for pelvic floor muscle training in prevention of POP

We found no primary prevention studies (interventions to stop prolapse from developing in asymptomatic women) of PFMT on POP. However, two studies focused on secondary prevention, as they included women who had not sought health care for assessment or treatment of the condition [37, 38] and a third published [39] 2-year follow-up of one of these [38] (Table 3).

**Table 3:** Short- and long-term effect of pelvic floor muscle training (PFMT) compared with no treatment or lifestyle advice on pelvic organ prolapse in the general female population (POP)

	Design/n	Prolapse	Training program	Drop-out/adherence	Outcome	Results	PEDro score (0–10)
Piya-Anant et al. -03 [40] Thailand	Unblinded RCT/654 women > 60 years	Anterior vaginal wall POP	PFMT (n = 330), vaginal palpation: 2 years of 30 contractions per day + eat more fruit and vegetables and drink 2 l of water per day. Ability to contract assessed by vaginal palpation. Follow-up every 6^th^ months Control (n = 324): no intervention, same follow-up	Drop-out: 28% Adherence: No report of PFMT adherence or change in fruit/vegetable intake	Primary outcome: No, mild or severe prolapse assessed by Valsalva on vaginal examination	PFMT: 27% worsening; Control: 72% worsening; p = 0.005. Effect only seen in women with severe prolapse No long-term follow-up	4
Ghroubi et al.-08 [41] Tunisia	RCT/ 47 women, mean age 53.4 (SD 11) No information about blinding	Stages I and II anterior vaginal wall POP	12 weeks, all had vaginal palpation PFMT (n = 27): 2 times per week for 5 weeks with individual PFMT + advice on healthy living by PT; home training 20 contractions per day for 7 weeks Control (n = 20): no treatment	Drop-out: 0 Adherence: not reported	Primary outcome: symptom of heaviness Secondary outcome; “Measurement of Urinary handicap scale” MUH; urodynamic tests; Ditrovie Quality of Life scale; patient satisfaction (VAS)	Heaviness after treatment: PFMT: 18.5%; Control: 70%, p < 0.001 Significantly better report on “MUH” in PFMT Uroflowmetry showed significant improvement in maximum flow rate **Two-year long-term follow-up:** benefits maintained in 20 of 27 (only 20 assessed) women in the PFMT group. No information on control group	6
Hagen et al.-09 [42] UK	Assessor blinded RCT/47 women, mean age 56 years (SD 9)	Symptomatic stages I and II POP (all types)	16 weeks 1: PFMT 5 visits with PT. Information of anatomy and PFMT + “the Knack” home exercise: 6 sets of max 10 contractions per day, use of diary + lifestyle advice sheet 2: Lifestyle advice sheet only	Drop-out: at 20 weeks: 13%, at 26 weeks: 15%. POP-Q data missing for 27/47 short term Adherence: 91% attended at least 3 PT sessions, 65% attended 5 visits. 61% rated as good/moderate compliers	Primary outcome: POP-Q; prolapse symptoms Secondary outcome: QoL/interference of daily living; self-report of change in POP; sexual function by ICIQ-VS, urinary and bowel symptoms by ICIQ-UI and ICIQ-BS Independent variable: Modified Oxford grading for PFM strength only in exercise group	PFMT significantly more likely to have improved POP stage (45% vs 0%, p = 0.04), significantly greater decrease in POP symptoms (3.5 vs 0.1, p = 0.021), significantly more likely to say their POP was better (63% vs 24%) Oxford grading (n = 15): significant improvement in exercise group: mean 0.5 (95% CI: 02–0.8). No effect on sexual function or urinary or bowel symptoms No long-term follow-up reported further than 26 weeks as above by questionnaire data	6
Brækken et al.-10 [25] a) and b) Brækken et al.-10 [43] Norway	Assessor blinded RCT/109 women, mean age 48.8 years (SD 11.8)	POP-Q stages I, II and III with and without symptoms. All types of POP	6 months, all had vaginal palpation and assessment of PFM strength and endurance PFMT: information on not to strain on toilet + ‘the Knack;’ 3 sets of 8–12 contractions per day, diary; weekly visits with PT for 3 months, every second week for 3 months Control: information on not to strain on toilet; ‘the Knack’	Drop-out: One drop-out in each group (1.8% of each) Adherence: 79% adhered to ≥ 80% of exercise sessions 86% attended all PT sessions and 89% adherence to home training program 10% control group reported that they had performed more PFMT than they did before baseline	Primary outcome: POP-Q; ultrasound of bladder and rectal position at rest; symptoms and bother (Mouritsen and Larsen-03) Secondary outcome ICIQ UI-SF; sexual dysfunction and bowel symptoms (Mouritsen and Larsen-03). Semi-structured interview on sexual function Independent variable: muscle strength by manometry	POP-Q stage: 11 (19%) in the PFMT vs 4 (8%) controls improved one stage (p = 0.04) Elevation of the bladder neck: ↑2.3 mm vs ↓0.6 mm; diff. 3.0 mm (95% CI: 1.5–4.4), p < 0.001 Elevation of rectal ampulla: ↑4.4 mm vs ↓1.1 mm, diff. 5.5 mm (95% CI: 1.4–7.3), p = 0.02 Symptoms: Vaginal bulging/heaviness: ↓ frequency 32/43 vs 8/26, p < 0.01; ↓ bother 29/43 vs 11/26, p < 0.01 ICIQ UI-SF. Effect size 0.62 in favor of PFMT, diff. 2.40 (95% CI: 0.90–3.80), p = 0.002 < 0.01 Bowel symptoms: no effect on emptying or solid FI Flatus: frequency diff. 31.2% (95% CI: 0.7–55) p < 0.01; bother diff. 25.3% (95% CI: 1.5–49.1) p < 0.01. Loose FI: frequency diff. 68.6% (95% CI: 40.2–97.0), p < 0.01; bother diff. 64.3% (95% CI: 39.2–89.4), p < 0.01 PFM strength (p < 0.01): PFMT: ↑13.1 cmH_2_O (95% CI: 10.6–15.5); Control: ↑1.1 cmH_2_O (95% CI: 0.4–2.7). Effect size: 1.21 Endurance (p < 0.01): PFMT: ↑107 cmH_2_O s (95% CI: 77.0–136.4); Control: ↑8 cmH_2_O s (95% CI: − 7.4–24.1). Effect size: 0.96 No long-term follow-up	8
Stüpp et al.-11 [44] Brazil	Assessor blinded RCT/37 women, mean age 55 years (SD 8)	Stage II POP. 56.7% had anterior vaginal wall POP, 10.8% posterior and 32.4% combination	14 weeks Group 1 (n = 21): PFMT 7 visits with PT. Use of quick pull of a vaginal cone and stretch reflex followed by active PFM contraction. Use of Knack during different tasks. Home exercise: 3 sets of 8–12 maximum voluntary contractions held for 6–10 s. PTs called patients every fortnight. Global stretching and lifestyle: weight loss, fluid intake, constipation, avoidance of heavy lifting Group 2 (n = 16): Control: taught how to perform PFM contractions with no protocol. Same lifestyle and global stretching as PFMT group	Drop-out: 0% Adherence: 100% in intervention and 76.2% in control. 91% adherence to home training program	Primary outcome: POP-Q Secondary outcome: Urinary and bowel symptoms by P-QoL Independent variable: Modified Oxford grading, PERFECT, sEMG	POP-Q stage: significantly greater improvement in training group (anterior vaginal wall: p < 0.001, posterior vaginal wall: p = 0.025) QoL: significant difference in favor of PFMT Symptoms: SUI significant difference in favor of PFMT (p = 0.002), straining to empty bladder (p = 0.031). No effect on vaginal bulge interfering with emptying bowel (p = 0.250) PFM variables: significant difference in favor of PFMT No long-term follow-up	5
Frawley et al.-12 [45] Australia ICS Abstract	Assessor-blinded multicenter RCT/168 women, mean age 55.9 years (SD 9.9)	Symptomatic POP-Q stage I, II, III. 80% had stage II POP, 73% had anterior POP	16 weeks PFMT: 5 appointments with PT, home exercise (see Hagen, 2009) + lifestyle advice Control: lifestyle advice	Drop-out at 6 months: PFMT – 14.3%; Control – 10.7%. Drop-out at 12 months: PFMT – 5.6%; Control – 21.3%. Adherence: 82.1% attended 4 or 5 PT sessions	Primary: prolapse symptom severity (POP-SS) Secondary: POP-Q stage Independent variable: Modified Oxford grading by vaginal palpation, manometry strength and endurance	Significant difference in symptoms in favor of PFMT at 6 and 12 months in favor of PFMT No difference between groups in POP-Q stage Significant difference in POP-Q points Ap and Bp at 6 months and Ap and Bp relative to hymen in favor of PFMT Significant better PFM strength in PFMT group measured by palpation at 6 months, but not at 12 months. No difference in manometry Long-term follow-up: 8 months with no supervised training or incentives as above	7
Kashyap et al.-13 [46] India	Unblinded RCT/140 women, mean age 47 years (SD 12)	POP-Q stage I-III. 63.5% had POP-Q stage I	24 weeks All had vaginal palpation Group A: one-to-one PFMT instruction + self-instruction manual. 6 follow–up visits at week 1, 3, 6, 12, 24. Home exercise one set of 10 contractions held for 10 s ×3 per day. Diary Group B: self-instruction manual + home training program. 3 follow-up visits at week 6, 18, 24	Drop-out: Loss to follow-up week 6: A: 27.1%, B: 12.9% Week 18: A: 22.9%, B: 31.4%. Week 24: Group A: 7.1%; Group B: 21.4% Adherence: not reported	Primary outcome: POP-Q stage POP symptom scale, VAS, QoL (PFIQ-7)	Significant improvement in POP-Q stage I and II and bulging at week 6, 18 and 24 in favor of PFMT. Significant difference between groups in favor of PFMT in VAS at week 18 and 24 Complete relief of symptoms in 24.5% in the PFMT group compared to 0 in the group B No long-term follow-up	6
Hagen et al.-14 [47] UK, NZ, Australia	Assessor-blinded multicenter RCT/447 women, mean age 56.8 years (SD 11.5)	Symptomatic POP-Q stage I, II, III 72% had stage II	16 weeks PFMT (n = 224): 5 appointments with PT + home exercise (see Hagen, 2009) and lifestyle advice Control: (n = 222): lifestyle advice	Drop-out at 6 months: PFMT: 16%; Control: 14% Adherence: 80% attended 4 or 5 PT sessions 40% responded to questionnaire at 18 months follow-up after cessation of training	Primary: POP symptom severity (POP-SS) Secondary: POP-Q, perceived change of POP, uptake of further treatment, cost-effectiveness, Sexual function: ICIQ-VS and PISQ-12 Urinary and bowel symptoms by ICIQ-UI and ICQ-BS	Significantly greater decrease in POP symptoms at 6 and 12 months in PFMT group (mean reduction in POP-SS from baseline 3.77 [SD 5.62] vs 2.09 [5.39]; adjusted difference 1.52, 95% CI 0.46–2.59; p = 0.0053) Symptom of bulging at 6 months: PFMT – 13.8% reduction in n; Control – 3.4% reduction in n Bulging at 12 months: PFMT – 20.5% reduction in n; Control – 17.0% reduction in n POP stage: PFMT – 20% improved; Control – 12% improved; p = 0.052 Uptake of further treatment: PFMT – 30%; Control – 55%; p < 0.001 No significant difference between groups in proportion with improved POP stage (27% vs 20%, p = 0.10) Significantly more likely to say their POP was better (62% vs 17% at 6 months, 57% vs 45 at 12 months) Uptake of further treatment: 24% PFMT, 50% Control (risk ratio 2.1, 1.5–2.9, p < 0.0001) Significantly better for aspects of bladder (UI: p = 0.01, ICIQ-UI: p < 0.001 and fecal urgency (p = 0.041 but not FI. Results diminished at 12 months Overall, the net cost of the intervention per woman was £170·24–£38·63 = £131·61 Adverse effect in 8 participants in the PFMT group unrelated to the intervention Long-term follow-up, see McClurg 2019	8
Wiegersma et al.-14 [38] Panman et al.-17 [39] same study, 2-year follow-up The Netherlands	Assessor blinded RCT/287 women, mean age 64.2 (SD 6.6)	Symptomatic POP-Q stage I and II	3 months Both groups had vaginal palpation. “Watchful waiting” or PFMT PFMT: Information of the pelvic floor. Individualized, not standardized following vaginal palpation, weekly visit to start with + home exercise	Drop-out: 26 in PFMT, 10 in watchful waiting; 87% completed follow-up Adherence: not reported	Primary outcome: POP-Q and overall symptoms Secondary outcome PFDI-20, PFIQ-7, PISQ-12 Independent variable: Vaginal palpation: normal, underactive, overactive, inactive	No difference in POP-Q. Significant improvement in PFDI-20 in PFMT vs control; 57% in PFMT vs 13% in control reported overall improvement in symptoms UDI-6 significant (0.007) but POPDI-6 (0.110) and CRAD-8 (0.165) not significant PFDI-urinary symptoms: sign difference in favor of PFMT: -6.0 (95% CI: -9.1 to -2.9), p < 0.001 No sign difference between groups in PISQ-12 or anorectal symptoms NB. Below the presumed level of clinical relevance (15 point) No effect of PFM variables Long-term follow-up:12 months follow-up of Wiegersma et al.-14 and 2 years Panman et a.-17 with no further incentives: PFMT greater pelvic floor symptom improvement, lower absorbent pad costs and was more effective in women experiencing higher symptom stress	5
Alves et al.-15 [48] Brazil	Assessor blinded RCT/46 postmenopausal women, mean age 65.9 years	Anterior and posterior POP on POP-Q, some women did not have POP	6 weeks All had vaginal palpation. All women performed a fitness program based on global muscle stretching, endurance and functional exercises PFMT: in addition to general program: 12 group sessions, 30 min, twice a week (four series of ten fast contractions together with four series of ten sustained contractions, lasting for 8 s) Control: general exercise program	Drop-out: 34.8% Adherence: not reported	Primary outcome: sEMG, Modified Oxford grading by vaginal palpation Secondary outcome: POP-Q, ICIQ	PFM contractility increased after PFMT, evaluated by sEMG (p = 0.003) and vaginal palpation (p = 0.001) Decrease in urinary symptoms (p < 0.001 for ICIQ-OAB scores p = 0.036 for ICIQ UI-SF) and anterior pelvic organ prolapse (p = 0.03) No long-term follow-up	6
Due et al.-16a) [49], Due et al.-16 b) [50], same study, b) 9-month follow-up Denmark	Assessor blind RCT/109 women, mean age 60 years (range: 33-79)	Symptomatic POP-Q ≥ stage II	3 months PFMT: one individual session with PT to learn PFM contraction, 6 group visits with PT, Knack + lifestyle + home exercise 3 sets of 10 contractions/day compared to Control: lifestyle: 6 group sessions	Drop-out: 18% at 3 months, 22% at 6 months Adherence: not reported 31/42 women completing the PFMT handed in their training dairies (74 %) 9 months after cessation of training: data available for 83/109, but not reported	Primary outcome: Global improvement scale, POP symptoms, POP-Q Secondary outcome: Sexual function by PISQ-12 Urinary and bowel symptoms by PFDI-20	Significantly better results in global improvement scale and POP symptoms in PFMT. No difference in POP-Q stage No effect on sexual function, urinary or bowel symptoms Long-term follow-up: 9 months after cessation of training; no difference between groups, but 13/43 (30%) control and 21/40 (52%) of PFMT group had not sought further treatment (p = 0.05)	7, 4
Ahadi et al.-17 [51] Iran	Pilot assessor blind RCT/40 women, mean age not reported	Stage I (42%) and II (58%) POP	12 weeks. Both groups did home PFMT for 12 weeks (10 repetitions of a 5-s squeeze followed by a 5-s release). In addition: 4 weeks of 1. PFMT and lifestyle advice in addition to biofeedback twice a week in clinics 2. Lifestyle advice sheet and PFMT without biofeedback at home only.	Drop-out: 2 = 5% Adherence: Not reported	Primary outcome: P-QOL questionnaire Secondary outcome: stage of POP by POP-Q Independent variable: manometry	P-QOL: Significant difference in favor of supervised PFMT No significant difference between groups in POP-Q No significant difference between groups in manometry assessment No complications No long-term follow-up	7
Hagen et al.-17 [37] UK, NZ	Assessor-blinded multicenter RCT 412 women, mean age 56.8 years (SD 11.5)	Women who had not sought help for POP Symptomatic POP-Q stage I, II, III 74.5% had stage II (all types)	16 weeks Intervention group: PFMT 5 appointments with PT over 16 weeks + home exercise (see Hagen, 2009) and lifestyle advice and Pilates-based pelvic PFMT classes and a DVD for home use + appointments at 1 and 2 years Control group: lifestyle advice leaflet (by post)	Drop-out: 21% at 1 year and 16% at 2 years (no difference PFMT vs Control) Adherence PFMT group: 74% attended 3 or 4 PT sessions Exercises last month (at 2 years): 77% PFMT vs 53% control group (odds ratio 3·22, 95% CI 1·94–5·32; p < 0·0001)	Primary outcome: POP-SS Secondary outcome: Sexual function by ICIQ-VS and PISQ-12, urinary and bowel symptoms by ICIQ-UI and ICIQ-BS, respectively	The mean POP-SS score at 2 years was 3·2 (SD 3·4) in the intervention group versus 4·2 (SD 4·4) in the control group (adjusted mean difference −1·01, 95% CI −1·70 to −0·33; p = 0·004) At 2 years, ICIQ-UI was significantly greater in the control group than in the intervention group (MD -0.83, 95% CI, -1.44 to -0.22; p = 0.008). Proportion of patients who had any urine leakage or severe incontinence did not differ between groups Bowel symptoms did not differ significantly between groups at 2 years, but interference associated with bowel symptoms was less frequent in the intervention group (MD -0.51, 95% CI -0.96 to -0.06; p = 0.026) Sexual symptoms did not differ between groups at 2 years 3 adverse effects all in intervention group (not related to PFMT): fall, pain in tailbone, chest pain and shortness of breath	6
McClurg et al. (ICS abstract-19) [52] Scotland	Multicenter RCT 8-10-year follow-up study of PFMT for Scottish part only (Hagen et al.-14)	See Hagen et al. 2014. POP stage I-III. No further incentives during follow-up time	See Hagen et al. 2014. No further incentives for PFMT during follow-up at 8-10 years Outpatient and inpatient hospital data linkage for subsequent treatment related to PFD (POP, UI, FI)	Drop-out: 310 of originally 447 participants from 11 of originally 23 centers Linked data available for 293 participants from the Scottish part of the original study only Adherence: not reported	Primary outcome: Any treatment relating to POP, UI, FI received during follow-up period Secondary outcome: treatment specifically for related surgery, conservative treatment (pessary or neurostimulation) or time to first treatment	Lower proportion of intervention group (43.6%) received treatment than the control group (52.8%). Difference was statistically significant in a mixed effect model (aOR 0.61, 95% CI 0.37 to 0.99 95% p = 0.047) and on multiple imputation (aOR 0.60, 95% CI 0.36 to 0.98, p = 0.042) Median time to first treatment or censoring in the intervention group was 3008 days (IQR = 589-3396) vs control group:2242 days (IQR = 628.5-3279. Significant hazard ratio in favor of the PFMT group: 0.65 (95% CI: 0.46-0.94, p = 0.020) No significant difference between groups in use of pessary or neurostimulation or undergoing any type of related surgery	Not applicable

In the unblinded RCT of Wiegersma et al. (2014) [38] PFMT was conducted individually over 3 months with a specialized physical therapist according to each participant’s PFM function assessed by vaginal palpation. Participants completed median 7 (range 5-9) visits with the physical therapist and were asked to do home exercises 3-5 times per week 2-3 times per day. They also received lifestyle advice to lose weight and improve toilet habits. In the assessor-blinded RCT by Hagen et al. (2017) [37] women were randomized to receive either printed lifestyle advice or 5 appointments with a physical therapist over 16 weeks and two blocks of once/week Pilates classes for 6 weeks. The Pilates class included specific PFMT in addition to the regular Pilates exercises

Both studies found statistically significant positive short-term effects on symptoms of POP, but not on POP-Q stage. Significant differences were maintained in some of the measures of symptoms at the 2-year longitudinal follow-up [39].

### Evidence for PFMT to treat POP in the peripartum period

Wu et al. (2018) [53] reported results from a systematic review of PFMT in the peripartum period for pelvic floor disorders and found 15 RCTs. Some of these studies reported on UI, AI and sexual dysfunction only and some also included electrical stimulation. Three RCTs [54–56] reported on PFMT for POP symptoms (dichotomous outcome; presence/absence of bothersome vaginal bulging) and were included in a meta-analysis. The authors concluded that there is very low-quality evidence that structured PFMT reduces POP symptoms at 6 to 12 months postpartum. Furthermore, they reported that there is moderate-quality evidence that structured PFMT did not change POP stage up to 12 months postpartum. The authors pointed out that evidence remains scarce on the long-term effects of structured postpartum PFMT on pelvic floor outcomes. A 12-year follow-up of an RCT comparing PFMT with control found no effect of PFMT on symptoms or prevalence of POP [57]. However, POP was not reported as an outcome in the original study [58].

In the secondary analysis of an assessor-blinded RCT with PFMT alone compared with only teaching and assessment of PFM strength for UI, 175 primiparous women with and without POP 6 weeks postpartum were included [54]. No effect was found in symptoms of bulging, bladder neck position or POP-Q stage after 4 months of group PFMT + everyday PFMT home training, starting at 6 weeks postpartum. The women diagnosed with POP had POP-Q stage I and II.

### Evidence for PFMT in treatment of POP in the general population

Table 3 shows RCTs assessing PFMT to treat anatomical POP or POP symptoms in the general female population. Eleven RCTs including women from 12 different countries were analyzed. Studies included POP of POP-Q stages I-III and results were generally reported for all stages together and not separately for each stage. Four studies compared PFMT with no treatment [40, 41, 44, 48]. The others compared PFMT with lifestyle intervention against lifestyle advice alone. Lifestyle advice included use of precontraction of the PFM before and during an increase in IAP (“the Knack”) and advice to avoid straining during defecation [25, 43] or general lifestyle advice only [42, 45, 47, 49]. Brækken et al. (2010a) [25] was the only study reporting on morphology and did not find any effect of advice to use “the Knack” on PFM strength or morphology. Adherence to the use of the Knack was not reported.

#### Quality of the interventions, dose response issues

There is some variation in the content of the PFMT interventions for POP in these trials. All the PFMT programs for POP included strength training of the PFM. In the study of Wiegersma et al. (2014) [38], the participants did strength training, but also many other exercise modalities and the chosen exercise program was up to the physical therapist with no standardization of the training protocol. All research groups included vaginal palpation to determine correctness of PFM contractions and some studies used additional biofeedback with the PFMT (Tables 3 and 4).

**Table 4:** Randomized controlled trials comparing pelvic floor muscle training (PFMT) and pessary for pelvic organ prolapse (POP)

	Design	Prolapse	Intervention	Drop-out/adherence	Outcome	Effect	PEDro score (0-10)
Manonai et al.-12 [59] India	Assessor blinded RCT 91 women, mean age 44 years (SD 8.9)	POP stage I and II	16 weeks: *Colpexin +PFMT *Colpexin sphere alone Vaginal assessment and Colpexin pull test every 4 week	Drop-out: 6; 6.6 % Adherence: 2 women in Colpexin + PFMT adhered to < 80% adhered to Colpexin use No further report of adherence to PFMT or Colpexin	PFM strength QoL POP stage	No change in POP-Q stage No difference between groups No long-term follow-up	8
Cheung et al.-16 [60] China	Assessor blinded RCT comparing PFMT alone with PFMT + pessary 276 women, mean age 62.6 years (SD 9.6)	Symptomatic POP stage I-III, all types. Mostly stage II (69% intervention vs 67% control) and anterior (64.7% intervention vs 66.4% control) on waiting list for surgery	16 weeks Intervention: Ring pessary (3 fit attempts) + PFMT: Nurse led: one teaching session, 3 individual sessions 4-8-16 weeks + daily home exercise daily with at least 2 sets of 8–12 preset exercise Repetitions per day, with 8–10 exercises per session at least two times per week Control: PFMT alone	Drop-out: Pessary + PFMT: 9.8% PFMT: 11.3% Adherence (at least 2 times a day/3 times/week at 12 months: Pessary + PFMT: 53.3% PFMT: 43.1% No information of pessary use	Primary outcome: change of POP symptoms and quality of life by PFDI-20; POPDI; PFIQ Secondary outcomes: *bothersome of prolapse symptoms *desired treatment *any complications	Sign higher decrease in POPDI/PFIQ in pessary group: PFDI-20: -29.7 compared with -4.7, P < 0.01; PFIQ: -29.0 compared with 3.5, P < 0.01) No difference in complication rates between groups. 66% were successfully fitted with pessary, 60% at 12 months De novo SUI: 48% in Pessary +PFMT vs 22.4% in PFMT alone No long-term follow-up	7
Panman et al.-16 [61] The Netherlands	Unblinded RCT on 162 women, mean age: 65.6 years (SD 6.4) with - 2-year follow-up	Symptomatic POP stage II (74%) and III (26%)	Two years Pessary + PFMT, median of treatments was 7 (IQR 6-10); over a median timeframe of 15.9 weeks (IQR 12-29.5 wk) (FUP 3-12-24 months) Group 1 (N = 82): pessary (open ring or ring with support or Gellhorn/Shaatz) fitted by trained research physician during 2 weeks (maximum 3 attempts) + appointments every 3 months Group 2 (N = 80): PFMT initial informative sessions + Same exercise regime (later tailored- based examination) 3-5 times a week, 2 or 3 times each day (during face-to-face contact and at home) + lifestyle and toilet advices + “the knack” +/- myofeedback or electric stimulation if needed	Drop-out: Pessary 8 (9.8%), PFMT 9 (11.3%) Did not receive randomized treatment: pessary 35 (34 unsuccessful fit); PFMT 4 Discontinued treatment: pessary 12, PFMT 10 Adherence: not reported	Primary: POP symptoms (PFDI-20, POPDI-6, PGI-I), POP stage (POPQ), costs (EuroQoL 5D 3L, QUALY, ICUR) Bladder and bowel symptoms (UDI-6, CRAD-8); specific and generic QoL (PFIQ-7, MOS-SF12, PCS-12, MCS12), sexual symptoms (PISQ12), adverse events Independent variable: vaginal palpation: normal, underactive, overactive or inactive	No difference between groups in overall PFDI-20 POP symptom score sign in favor of pessary (mean difference -3.2 points [95% CI, -6.3 to -0.0; P = 0.05]) Cost over 2 years PFMT: 437 $ Pessary:309 $ Pessary fitting fails in considerable portion of women Pessary treatment associated with more side effects than PFMT	6

*AI*, anal incontinence; *A-QoL*, Assessment of Quality of Life; *C*, Control; *CG*, control group; *CRADI-8*, Colorectal-Anal Distress Inventory-8; *EMG*, electromyography; *EURO-QoL 5D-3L*, EuroQol five dimensions-three-level Quality of Life Instrument; *FSFI*, female sexual function index; *I*, intervention; *IG*, intervention group; *ICIQ-UI-SF*, International Consultation on Incontinence Questionnaire-Urinary incontinence short form; *ICIQ-VS*, International Consultation on Incontinence Questionnaire Vaginal Symptoms; *ICIQ-BS*, International Consultation on Incontinence Questionnaire-Bowel symptoms; *ICIQ-FLUTS*, International Consultation on Incontinence Questionnaire-female lower urinary tract symptoms; *IIQ-7*, Incontinence Impact Questionnaire-7; *ISI*, Incontinence Severity Index Questionnaire; *LAG*, Lifestyle advice group; *MCS-12*, Mental Component Summary-12; *MOS-SF12*, Mental Health Component Summary-Short Form-12; *NMES*, neuromuscular electrostimulation; *PCS-12*, Physical Components Summary-12; *PFMT*, pelvic floor muscle training; *PISQ-12*, Pelvic Organ Prolapse Incontinence Sexual Questionnaire-12; *PFDI-20*, pelvic floor distress inventory questionnaire-20; *PFIQ-7*, Pelvic Floor Impact Questionnaire-7; *PGI-I*, Patient Global Impression of Improvment; *POP*, pelvic organ prolapse; *POP-Q*, Pelvic Organ Prolapse Quantification System; *POPDI-6*, Pelvic Organ Prolapse Distress Inventory-6; *POP-SS*, Pelvic Organ Prolapse Symptom Score; *P-QoL*, Prolapse Quality of Life Questionnaire; *PT*, physical therapist; *QUALY*, quality-adjusted life year; *ICER*, incremental cost-effectiveness ratio; *RCT*, randomized controlled trial; *sEMG*, surface EMG; *SF*, sexual function; *SF-6*, 6-Item Short Form Health Survey; *SF-12*, 12-Item Short Form Health Survey; *SF-36*, 36-Item Short Form Health Survey; *SUI*, stress urinary incontinence; *TPUS*, transperineal ultrasound; *TTNS*, transcutaneous tibial nerve stimulation; *UDI-6*, Urinary Distress Inventory 6; *UDI-19*, Urogenital Distress Inventory-19; *UI*, urinary incontinence; *VAS*, visual analog scale; *WHOQoL*, World Health Organization quality of life questionnaire

The training period varied between 4 weeks and 2 years, and number of visits with the physical therapist varied between 4 and 18. PFMT was taught individually in all but one trial [49], who did group training. Supervised PFMT was always combined with a home training program. Drop-outs were low and adherence high in most studies. The highest number of visits with the physical therapist was in the study of Brækken et al. (2010a, 2010b) [25, 43], which included 18 visits over 6 months. No studies compared different training dosages. Overall, more intensive and supervised programs showing better results, both for symptoms and POP-Q stage of POP.

For short-term effect of PFMT, the RCTs showed PFMT to be effective in treating POP, demonstrating statistically significant improvement in POP symptoms [41–46, 48, 49] and/or anatomical POP, assessed by POP-Q stage [40, 42–44, 46, 48] compared to the comparison group. The results typically show that PFMT can improve POP-Q exams by one stage (from I to 0, II to I and III to II). Frawley et al., (2012) [45], Due et al. (2016) [49] and Ahadi et al. (2017) [51] did not find a significant change in POP stage, but significant improvements were found in some of the individual POP-Q measurements (POP-Q points Ap and Bp) in the study by Frawley et al. (2012) [45]. Methodological scores on PEDro ranged from 4 to 8, with five of the studies scoring 7–8 (Table 3).

Table 4 reports RCTs that compared PFMT with a pessary. Three RCTs were found, but the studies used different pessaries and different PFMT protocols. One study did not find any differences in outcome between PFMT and PFMT + pessary [59] while two studies found significant differences in favor of pessary use. Both Panman et al. (2016) [61] and Cheung et al. (2016) [60] found that pessary fitting fails in a considerable number of women; 40% reported at 12 months [60]. De novo stress urinary incontinence was higher in the pessary group than in the PFMT group, 48% vs 22%, respectively [60].

### Evidence for PFMT pre- and post-POP surgery

Table 5 shows the published RCTs on PFMT in connection with POP surgery. Eleven full publications or meeting abstracts which were published from 2005 until 2021 [62–72] were included. We excluded one study because of inclusion of SUI and POP surgery as well as lack of reports of symptoms or stage of POP [66]. PFMT started preoperatively in all studies with between one to four sessions and continued with individual visits post-surgery.

**Table 5:** Evidence for pelvic floor muscle training (PFMT) in connection with pelvic organ prolapse (POP) surgery

	Design	POP	Exercise intervention	Drop-out/adherence	Outcome	Results	PEDro score (0-10)
Frawley et al.-10 [65] Australia	Assessor blinded RCT 58 women, mean age 56.5 (SD 10.5)	Women of any age undergoing vaginal or laparoscopic-assisted vaginal surgery for either POP repair (primary or recurrent) and/or hysterectomy (94% POP surgery)	10 months Intervention (N = 30): PFMT+ lifestyle advice + the “knack” during 8 sessions with PT. 1 preoperatively (set of 6–8” contractions, with a rest period in between each contraction, repeated 8–12 times, 3 times/day; variety of positions) and 7 postoperatively at 3d-7w-7w-8w-10w-12w and 9 months (reduction dosage/intensity immediate postoperative period and gradual increase to the preoperative intensity by 6 weeks postoperatively and maintenance of an intensive level of PFMT for 3–6 m, then reduction in frequency of exercise sets to 1–2 sets/day by 12 month) Control (N = 28): pre- and postoperatively advice by gyn and nurse+lifestyle advice +/- PFM exercise without PT supervision	Drop-out = 12.1% (3 in intervention, 4 in control) Did not receive allocated group: 6 intervention + 1 control Adherence: *89% to physiotherapy visits *67% reported home training: 22% at < 1/week, 23% training 1–6 days/week, 22% training daily	Primary: Prolapse symptoms (obstructive symptoms of POP as detailed in the UDI) Secondary: bladder symptoms (UDI-19), QoL (IIQ-7, AQoL), bowel symptoms (Wexner, Constipation Scoring System), 3-day bladder diary, 48-h pad test Independent variable: Modified Oxford grading by vaginal palpation and manometry	12-month postoperative no difference in prevalence of the primary outcomes (ORs 1.2, 1.3). No significant differences between groups on change scores of UDI (mean: 44.1 [5.1]; 54.0 [5.4], P = 0.20) nor the IIQ (median: 0.0 [9,14]; 10.0 [5,19], P = 0.09) PFM strength significant in favor of intervention on (vaginal testing and manometry) No further follow-up	6
Pauls et al.-13 [73] Pauls et al.-14 [71] Same study US	Assessor blinded RCT N = 57 women, mean age: 58 (SD 10.5) years	Symptomatic POP (all types) undergoing reconstructive POP surgery	12 weeks Long term at 24 weeks = 3 months after end treatment) Intervention (n = 29): PFMT 2 weeks preoperatively and 2-4-6-8 and 12 weeks postoperatively, in conjunction with a physician assessment Control (n = 28): physician assessment alone at all postoperative intervals	Drop-out: 5 PFMT vs 3 Control Adherence: not reported	Primary: WHOQOL-BREF Secondary outcome: POP symptoms (PFDI 20), POP stage (POPQ), bladder and bowel symptoms (voiding diaries, PFDI20), QoL (PFIQ7, SF-12), sexual symptoms (FSFI, PISQ12) Independent variables: Modified Oxford grading by vaginal palpation, intravaginal sEMG	At 12 weeks (Pauls et al.-13) No statistically significant difference between groups in POP symptoms, QoL or sexual, urinary or bowel symptoms POP stage not shown PFM variables: no difference in Modified Oxford grading between groups, significant difference in sEMG at rest at 12 weeks in favor of PFMT Long- term follow-up at 6 months after surgery (Pauls-14): no difference between groups	5, 5
Barber et al.-14 [62] US	Assessor blinded multicenter, 2 × 2 factorial RCT (randomizations: 1: perioperative PFMT or usual care; 2: SSLF or ULS) N = 374 women,mean age: 57.5 years (SD 10.9) PFMT vs 56.9 years (SD 10.9) Control	Stage II (39%), III (57%) or IV (4%) symptomatic POP (all types) + SUI undergoing POP surgery.	12 weeks PFMT (n = 186): 5 visits. First 2-4 weeks before surgery (45 contractions/day, initial contraction 1 – 3,” increasing 1-2” per week to a maximum of 7” and instructions for resuming exercises postoperatively). 4 visits after surgery (2, 4-6, 8, 12 weeks). Individualized progressive PFMT (maximum 45–60 contractions per day and maximum 10 s of each contraction) + educational behavioral strategies. Maintenance program of 15 contractions per day at the maximum contraction duration achieved Control group (n = 188): Usual care: routine perioperative teaching and standardized postoperative instructions	Drop-out: 6 months; 6 PFMT vs 8 Surgery alone 24 months: 34 (18.3%) PFMT vs 24 (12.8%) surgery alone p = 0.15. Adherence: 93.4% (6 months) and 81,4% (24 months) to home training program 4.3% of surgery group received supervised PFMT outside of the study (24 months)	Primary outcome surgery: POP stage (POP-Q), POP symptoms (POPDI-6, PFDI-20, PGI-I), anatomic failure as composite outcome of position of organs + retreatment (1)descent of the vaginal apex more than one-third into the vaginal canal or (2) anterior or posterior vaginal wall descent beyond the hymen or (3) retreatment for prolapse) PFMT: primary outcome at 6 months: UDI and at 2 years: POPDI and anatomic success Independent variables: Vaginal palpation by Brink grading Adverse events	PFMT was not associated with greater improvements in POP scores than control group [treatment difference −8.0 (95% CI −22.1, 6.1) at 24 months] PFMT was not associated with greater anatomic success (24 months) PFMT was not associated with greater improvements in urinary or bowel scores (6-12-24 months) than control group or in QoL scores No differences in PISQ-12 or body image scores between groups at any point Baseline PFM strength mean score: 8 (Brink range 3–12). At 24 months, mean Brink scores were 8.2 and 8.0 in PFMT and usual care groups respectively (p = 0.27) Long-term effect: as above for 2 years after surgery; no effect Severe adverse events: Surgery: 16.5% and 16.7 in each surgery group PFMT: no adverse events reported	6
Weidner et al.-17 [72] US	Same as Barber et al. 2014	See Barber et al.-14	See Barber et al.-14	See Barber et al.-14	Secondary outcome of Barber et al.-14: Primary outcome of present study: change in body image and in Pelvic Floor Impact Questionnaire (PFIQ) short-form subscale, 36-item Short-Form Health Survey (SF-36), Pelvic Organ Prolapse-Urinary Incontinence Sexual Questionnaire short form (PISQ-12), Patient Global Impression of Improvement (PGII) Independent variable: Vaginal palpation by Brink scores	No statistically significant differences between groups in PFIQ, SF-36, PGII, PISQ-12, body image scale measures or Brink score	4
McClurg et al.- 14 [69] UK	Multicentre feasibility pilot RCT 57 women, median age: 60 (range 35–80) years	Symptomatic POP Stage I (8%), II (63%), III (27%), (50% anterior POP) undergoing primary POP surgery	12 weeks 1 pre- and 6 postoperative sessions PFMT (n = 28): Preoperative: information of PFM by PT+ “the knack” + daily PFMT consisting of 3 sets of 10 maximum contractions (up to 10-s hold)/day with 4 s rest between sets, following by a 1-min rest followed by 10 fast contractions + lifestyle advice Same sessions postoperative Control (n = 29): only lifestyle advice postoperative	Drop-out: 7% PFMT and 7% control after surgery, 18% (50% examination) intervention and 14% (72% examination) control 6 months; 50% (82% examination) intervention and 56% (83% examination) control 12 months. Numbers attending for assessment were TG = 14/28 (50%) and CG = 11/29 (28%); at 12 months, 5/28 (18%) and 5/29 (17%) attended Adherence: Intervention group attended 80% of treatment sessions. Home exercise: overall good adherence in those recording (but only 28% recorded)	Primary outcome: POP symptoms score (POP-SS) Secondary outcome: POP stage (POP-Q) Bladder (ICIQ-IU-SF) and bowel (ICIQ-BS) symptoms, sexual symptoms (PISQ-12), QoL (SF-12) Independent variables: Modified Oxford grading by vaginal palpation, adverse events	Significant difference in POP-SS between groups in favor of PFMT at 12 months [MD 3.94; 95 % CI 1.358–6.750; t = 3.248, p = 0.006) Significant difference in SF-12 in favor of PFMT at 12 months (MD −6.88333; 95% CI −11.344 to −2.431, t = −3.218, p = 0.004) No differences in ICIQ or PISQ-12 scores POP stage changes and PFM variables no interpretation due to missing data (only 10 women examined at 12 months) Long-term follow-up: 6 and 12 months after surgery; as above No adverse events reported	7
Jelovsek et al.-18 [67] (follow-up of Barber et al.-14 [62] and Weidner et al.-17 [72]	5-year follow-up of OPTIMAL trial, see Barber et al. 2014 and Weidner et al. 2017	See Barber 2014 and Weidner et al.-2017	See Barber et al. 2014 and Weidner et al. 2017	374 originally participated At 5 years: 244 completed the extended trial Adherence: no report of adherence at 5 years or during time elapsed from surgery	See Barber et al.-14Primary surgical outcome: POP-Q and symptoms of bother Primary PFMT outcomes: time to anatomic failure and Pelvic Organ Prolapse Distress Inventory scores (range: 0-300)	Long-term follow-up 5 years after surgery: no differences between surgery with PFMT and surgery without neither on primary or secondary outcomes. No change since 24-month follow-up Estimated surgical failure rate was 61.5% and 70.3% in the two surgical groups Estimated anatomic failure rate was 45.6% in the PFMT group and 47.2% in the usual care group (adjusted difference, −1.6% [95% CI, −21.2 to 17.9]) Improvements in Pelvic Organ Prolapse Distress Inventory scores were −59.4 in the BPMT group and −61.8 in the usual care group (adjusted mean difference, 2.4 [95% CI, −13.7 to 18.4])	Not applicable
Brandt & Janse Van Vuuren-20 [63] South Africa	Double blind 3-arm RCT 81 women, 18-75 years	POP Stage I-III scheduled for reconstructive POP surgery	3 months 4 sessions with PT in group 1 and 2 within 6 months 1. PFMT including vaginal palpation, ultrasound, observation and sEMG of contraction 8-10 contractions once 5 days a week in progressive positions 2. PFMT + abdominal training (TrA) 3. Usual care, taught to pre-contract the PFM, no clinical assessment	Drop-out: 8.6% 2 in PFMT 2 in abdominal training 3 in control Adherence: 60% PFM exercises at 3 months, 55% at 6 months. Non-sig difference between group 1 and 2	Primary: The Prolapse Quality of Life questionnaire, two-dimensional ultrasound, PERFECT (palpation: power, endurance, repetitions, fast contractions, every contraction timed), sEMG, Sahrmann scale, and manometry Urinary frequency at 3 months, bulging at 6 months Discomfort at 6 months LA at 6 months Sahrmann 6 months Secondary: *adherence *lumbar and pelvic health	No effect on Prolapse Quality of Life or POP symptoms PFMT: Significant Improved power, number of fast contractions, amount of movement, endurance, and Sahrmann and manometry measures – compared with the control group. PFMT + Abdominal training: Significant improvement in number of PFM contractions, Sahrmann and manometry measures compared with the control condition Significant (p < 0.05) increase in bulging and discomfort Both groups showed significantly increased urinary frequency (p < 0.05) compared with control No adverse effects of either interventions No report of long-term effect above 6 months	8
Duarte et al.-20 [64] Brazil	Assessor blind RCT 96 women, age: 66.7% > 60 years, 28.1% 40-60 years, 5.2% 37-40 years	Symptomatic POP II (43%), III, IV (all types) undergoing POP surgery	9 weeks Both groups had vaginal palpation and manometry PFMT (n = 48): 4 preoperative (within 2 weeks preoperatively) and 7 postoperative supervised sessions with PT Postoperative PFMT started at day 40 after surgery with 7 postoperative weekly sessions Each session: 4 sets of 10 repetitions of maximum voluntary contractions with a 7 s hold/7 s rest (supine, sitting, kneeling and standing) + 5 quick contractions Same protocol at home at least 3 times/week Control (n = 48): only surgery	Drop-out: 2 (4%) in PFMT, 0 in control Adherence: 93% Intervention > 75% of the supervised sessions. 76% home training on 75-100% of the days prescribed	Primary outcome: POP symptoms (PFDI-20, PGI-I), Secondary: bladder and bowel symptoms, QoL (PFIQ-7), sexual symptoms (PISQ-12), perception of improvement Independent variables: PFM strength and endurance by manometry	No substantial difference in POP symptoms between the intervention and control at day 40 (adjusted mean difference -6, 95% CI -25 to 13) or day 90 (adjusted mean difference -4, 95% CI -23 to 14) The experimental group perceived marginally greater global improvement than the control group [mean difference -0.4 (95% CI -0.8 to -0.1) at day 90] PFMT was not associated with better improvements in urinary, bowel, sexual or QoL scores than control group No difference in PFM variables between groups No report of further long-term effect	8
Nyhus et al.-20 [70] Norway	Assessor blind RCT 159 women, mean age: PFMT+ surgery 60.1 (SD 11.2) vs surgery: 60.6 (SD 10.9)	Symptomatic POP II, III, IV (60% ≥ III) (all types) undergoing POP surgery	Mean 22 weeks waiting for surgery PFMT (n = 81): Preoperatively: vaginal palpation + written information leaflet and asked to daily PFMT consisting of 8–12 contractions, each held for 6–8 s, three times a day + lifestyle advice + “the knack” Two personal visits with PT at 2 and 6 weeks after inclusion preoperatively including vaginal palpation Optional weekly PFMT in groups with PT Control (n = 78): only surgery	95% women completed the study intervention = 75, controls = 76) Drop-out: 6 in Intervention vs 2 in Control Adherence: 60 (80%) women in the PFMT group adhered to ≥ 70% to the intervention None of the participants met for the voluntary weekly group training sessions	Primary: POP symptoms (bulge sensation, VAS), POP stage (POP-Q), position of organs and muscle morphology (ultrasound 3D -TPUS) Secondary: anatomical POP Independent variables: Modified Oxford grading by vaginal palpation, 3D-TPUS, manometry, sEMG	No difference was found between surgery + PFMT and control with respect to PFM contraction assessed by Modified Oxford grading: 2.4 vs 2.2;P = 0.101), manometry (19.4 vs 19.7 cmH_2_O; P = 0.793), surface EMG (33.5 vs 33.1 mV; P = 0.815) and ultrasound (change in hiatal APD, 20.9% vs 19.3%; P = 0.211). Sensation of vaginal bulge: no difference between groups (VAS, 7.4 vs 6.0 mm; P = 0.598), POP distance from the hymen in the dominant prolapse compartment (−1.8 vs−2.0 cm; P = 0.556) and sonographic descent of the bladder (0.5 vs 0.8 cm; P = 0.058), cervix (−1.3 vs −1.1 cm; P = 0.569) and rectal ampulla (0.3 vs 0.4 cm; P = 0.434) No further long-term effect reported	7
Mathew et al.-21 [68] Norway	RCT/159 women >18 yrs Same study as Nyhus et al. 2020	See Nyhus et al. 2020	See Nyhus et al. 2020.	See Nyhus et al. 2020	Primary outcomes: Symptoms of urinary and colorectal-anal distress assessed by validated PFDI sub-scales: UDI-6 and CRADI-8. Secondary outcome: patient reported quality of life related to urinary and colorectal-anal symptoms using the PFIQ sub-scales: UIQ and CRAIQ	No added effect of preoperative PFMT on symptoms or QoL related to urinary and colorectal-anal distress: no difference in mean postoperative symptom and QoL scores (95% CI) between the intervention and control group: UDI-6 16 (12–21) vs. 17 (13–22), CRADI-8 15 (11–18) vs. 13 (10–16), UIQ 11 (7–15) vs. 10 (6–13) and CRAIQ 5 (2–7) vs. 6 (4–9), all p > 0.05	

Only one of the RCTs showed any benefit of adding PFMT to surgery for POP [69]. The latter was a pilot feasibility study with 67 participants randomized to either PFMT or usual care in connection with surgery and showed that the training group had fewer prolapse symptoms at 12 months.

### Evidence for PFMT in women with POP on sexual dysfunction, bladder and anorectal symptoms

The results of PFMT on sexual dysfunction, bladder and anorectal symptoms for women with POP are presented in Tables 3, 4 and 5. None of the studies had sexual function or bladder and anorectal symptoms as the primary outcome. The size of the studies varied from 57 [71] to 374 participants [72]. One study included women with POP-Q stage I, II and III, regardless of POP symptoms [74], while the others included women with symptoms.

#### Sexual dysfunction

Tables 3, 4 and 5 include studies reporting the effect of PFMT on sexual function in women with POP. Two studies found some effect of PFMT on sexual function [74, 75]. The short-term improvement of Pelvic Organ Prolapse Incontinence Sexual Questionnaire-12 (PISQ-12) and Female sexual function index (FSFI) scores described by Pauls et al. (2013) [75] was lost after 24 weeks [71]. Braekken et al. (2015) [74] using a validated questionnaire from Mouritsen et al. (2003) [73] found no significant change in the number of sexually active women and no significant differences between groups regarding change in satisfaction with frequency of intercourse. However, interview data showed that 19 (39%) of women in the PFMT group reported improved sexual function compared to only two participants (5%) in the control group (*P* < 0.01). Some participants reported increased control, strength and awareness of the pelvic floor, improved self-confidence, sensation of a "tighter" vagina, improved libido and orgasms, resolution of pain with intercourse and heightened sexual gratification for partners. Additionally, the study found that participants who described improved sexual function obtained the greatest increases in pelvic floor muscle (PFM) strength (mean 16 ± 10 cmH_2_O) and endurance (mean 150 ± 140 cmH_2_O s) (*P* < 0.01).

#### Bladder and bowel symptoms

The results related to PFMT on bladder and bowel symptoms in women with POP are shown in Tables 3, 4 and 5. Eight studies found that PFMT improved bladder symptoms [38, 39, 43, 44, 47, 60, 61, 74] and five studies found improvement of anorectal symptoms [39, 43, 44, 47, 61, 74]. Braekken et al. (2010b) [43] and Braekken et al. (2015) [74] report from the same RCT using different outcome measures.

### Long-term effect of PFMT in treatment of POP

We defined “long term” as follow-up of at least 6 months after cessation of the intervention conducted in the original RCTs. There are a limited number of studies on the long-term effects of PFMT for POP (Tables 3, 4 and 5). The follow-up period varied between 6 months and 8 to 10 years, and there were no reminders or follow-up of PFMT during the follow-up period. Long-term effect was found in the findings of Panman et al. 2017 [39] and McClurg et al. 2019 [52], but not of Barber et al. 2015 [62], Jelovsek et al. 2018 [67] and Due et al. 2016b [50]. The studies with no long-term effect did not show any short-term effect.

Adverse effects of PFMT in women with POP are almost non-existent, and, if present, mostly not related to the PFMT.

## Discussion

This narrative review found no primary prevention RCTs on PFMT for POP and sparse evidence on early interventions in the general female population and the peripartum period. PFMT has been shown to significantly improve POP symptoms and associated pelvic floor complaints. Anatomic POP has been shown to improve by one POP-Q stage. It is unclear how clinically relevant this is because there are insufficient data describing the elevation of the leading edge of prolapse from beyond to above the hymen with PFMT. Studies included women with POP-Q stage I to III POP, and the effect was generally reported for all stages together, and not separated between stages, possibly because of low sample sizes. One pilot feasibility RCT showed significant benefits of PFMT as an adjunct to surgery but this was not proven in the nine other studies. Long-term data are sparse, and results are inconsistent.

### Prevention and early peripartum intervention

Given the anatomical and biomechanical rationale and evidence for PFMT to change pelvic floor morphology [25], it is theoretical to assume that PFMT would be effective in prevention of POP. Regular PFMT and early intervention to stop development and worsening of POP (primary prevention) would probably be the best health care investment to reduce suffering and cost of this prevalent female condition. If PFMT is provided as group training, it could be more cost-effective than individual training [22]. However, evidence from robust RCTs in prevention of POP is lacking. Furthermore, RCTs on PFMT in prevention of POP should optimally proceed over 20–30 years and include one PFMT training and one non-training group. It would be costly, time-consuming and probably not possible to keep women adhering to an exercise training program for such long time, nor would asking women not to do PFMT be ethically acceptable. The two included studies on secondary prevention in our review [37, 38] could have been classified as treatment as some of the included women had symptoms of POP, but as the participants had not sought help for POP, we chose to classify them as preventive studies. The results of these RCTs showed some benefit, but the effects were not that convincing [37, 38]. Lack of large effect sizes can be attributed to low training dosage (mode of exercise, frequency, intensity, duration and adherence to training) [76]. The idea of including correct PFMT into general exercise classes for women would probably be the only option to enable women adhere to regular PFMT for the purpose of preventive exercise [22]. However, that would only apply for women who are regularly physically active, and many of them would exercise independently and not in exercise classes.

Somewhat surprisingly, studies have found that POP is also prevalent in nullipara women [77]. As vaginal birth is a main risk factor for the condition, PFMT should be offered at an early stage either during pregnancy or after childbirth [78]. However, since there is insufficient evidence to suggest this based on results from RCTs, this would be based on expert opinion only. Some of the studies in the systematic review of Wu et al. (2018) [53] on postpartum PFMT for PFD were not registered in PubMed [55, 56, 79, 80], and only three RCTs were included in the meta-analysis of PFMT for POP. Three of the RCTs included electrical stimulation in addition to PFMT [79, 81]. The inclusion of these confounders means that we cannot conclude the effect of PFMT alone in these postpartum studies. The conclusion of Wu et al. (2018) [53] is that there is very low-quality evidence that structured PFMT reduces POP symptoms at 6 or 12 months postpartum, which is based on the two RCTs not found in PubMed [55, 56]. Additionally, the evidence for a positive effect in the postpartum period is not supported by results from Bø et al. (2015) [54] or the 12-year follow-up of conservative management of postnatal urinary and fecal incontinence and POP outcomes by Glazener et al. (2014) [57]. The training dosage and level of supervision might have been too low in the Glazener study (2001) [58] but was quite high in the study by Bø et al. (2015) [54]. However, the primary outcome of Bø et al. [54] and Glazener et al.’s [58] RCTs was UI, and both studies included women with and without POP; thus, the sample size may have been too small to detect significant differences for POP. In addition, in the Bø et al. RCT [54], a substantial number of the participants had major levator ani tears, which may have made it more difficult to achieve positive results. Higher IAP with lifting of a newborn infant and baby equipment may further explain difficulties in achieving positive effect of PFMT in the postpartum period. Further RCTs are warranted to investigate the effect of early prevention and treatment using PFMT on women with symptomatic POP in the peripartum period.

### General population

The effect of PFMT in the general female population with POP showed overall positive short-term results on POP symptoms, and in most of the studies measuring anatomical POP, the prolapse improved one stage [40, 42–44, 46, 48]. The studies with no effect on POP-Q stage had a lower training dosage compared with studies finding statistically significant improvement in anatomical POP [45, 49, 51]. Interventional quality of exercise training studies is dependent on training dosage [22, 76]. We did not find any RCT comparing different training dosages. Ahadi et al. (2017) [51] claimed to compare PFMT with or without biofeedback but added 4 weeks of PFMT with biofeedback to home exercise. Hence, this is not an equivalent comparison. For UI, no additional effect of adding biofeedback was found in an RCT with equal dosage and supervision of PFMT [82]. Kashyap et al. (2013) [46] compared six visits over a 6-month training period using a self-instruction manual only to one-on-one PFMT plus instruction manual, and the results showed the same overall beneficial effect on symptoms and POP-Q stage in the one-on-one PFMT group as Brækken et al. [25, 43]. As for all exercise training programs, this review found that there is a dose-response relationship with more intensive and supervised programs showing better overall results, both for symptoms and POP-Q stage of POP.

This review found only three RCTs comparing PFMT with a pessary, highlighting the need for further high-quality RCTs for this comparison. The level of evidence for pessaries as an effective intervention for POP was level three evidence in the 2017 ICI systematic review [26] and this corresponds with the latest Cochrane review [83]. As wearing a pessary over time may unmask occult stress urinary incontinence, this should not be considered a complication of pessary use [84].

### Sexual, bladder and anorectal comorbidities

Many of the RCTs on PFMT in women with POP also evaluated the effect on comorbidities such as sexual dysfunction, bladder and anorectal symptoms and found positive results. However, none of the studies had these comorbidities as primary outcomes and the studies included women with and without these symptoms. The power of the studies not finding an effect may be too low for these secondary analyses. Most studies used general validated instruments for the overall conditions, and more detailed information on the effect of UI and AI was not always available.

Sexual dysfunction in women is frequently associated with pelvic organ prolapse (POP) [85]. In a systematic review [86], the authors found only Level 2 evidence that PFMT can improve women's sexual function, and the literature is scarce about the effect of PFMT on sexual function in women with POP. As for other comorbidities, the training duration in some of the studies was short because it was done as an adjunct to surgery [64, 71], had a limited number of supervised sessions which may have limited adherence to the training protocol [49, 72] and included women both with and without sexual dysfunction. There is some evidence that women without sexual dysfunction have increased PFM strength compared to women with sexual dysfunction [87]. However, sexual function is influenced by several interpersonal, contextual, personal, psychological and biological factors including POP. Partner factors may adversely affect sexual function and diminish the benefit of PFMT [86]. We found that only two RCTs which showed some positive effects of PFMT on sexual function of women with POP [71, 74]. There is need for new RCTs specially designed to investigate the effect of PFMT in specific types of sexual dysfunction in women with POP.

Some of the studies assessing bladder and anorectal symptoms had a relatively short duration of the PFMT protocol as they were investigating perioperative PFMT [64, 71, 75] or a limited number of supervised sessions that could have limited the adherence to training protocol [37, 42, 47, 49, 69, 72]. The two RCTs offering supervised PFMT for a longer time showed improvement in bladder and anorectal symptoms in women with POP [25, 39, 74]. Future high quality RCTs are therefore still needed to investigate the impact of intensive supervised PFMT protocols on bladder and anorectal symptoms in women with POP.

### Effect of PFMT pre- and post-POP surgery

It is somewhat counterintuitive that PFMT does not seem to be effective pre- and post-POP surgery. This is despite evidence that it is effective in the treatment of POP [26]. However, women included in surgical studies may be a selected group with more severe POP resulting from major PFM tears, perineal tears or higher hiatal area ballooning. In addition, it may be difficult to show additional effect of PFMT if surgery alone is effective, and a possible effect of PFMT may only be expected to be seen after many years of continuous PFMT. The longest follow-up period was 5 years after surgery, but there were no incentives for continuous PFMT during the follow-up period after the last training session 12 weeks post-surgery, and there was no report on adherence to maintenance training [67]. Furthermore, there was no effect of the original training program in this study [62], and a long-term effect may therefore not be expected. The lack of effect of PFMT in connection to POP surgery warrants that such studies should be analyzed separately from treatment trials in the general population and not be merged in meta-analyses.

Furthermore, the recurrence rate following traditional surgery for POP is approximately 30% [88, 89], and surgery for prolapse can be associated with various adverse events and potentially poorer quality of life than the original condition [90, 91]. Mesh is one option for surgical treatment of stress urinary incontinence (SUI) and POP. However, some women experience serious complications [88, 89], and mesh is now the subject of intense global scrutiny after reports of serious negative long-term effects [92]. The National Institute for Health and Care Excellence (NICE) Guidelines (2019) [27] state that there is some evidence of benefit for all surgical procedures for POP recommended in their guideline, including mesh procedures, but limited evidence on long-term effectiveness and adverse effects. They conclude that “the true prevalence of long-term complications is unknown.” Although the rate of severe complications may be low [90], due to high surgery rates, a substantial number of women will suffer after POP surgery worldwide [92]. Non-invasive treatments, such as lifestyle interventions and PFMT, should therefore be considered before surgical interventions [26, 27].

### Long-term studies

There are general challenges to long-term studies of any intervention due to loss-to follow-up, co-interventions, treatment switching and competing events [93, 94]. For exercise training there are additional challenges such as non-adherence to maintenance training [93, 94]. In a systematic review of long-term effect of PFMT for SUI/MUI 19 studies with follow-up ranging from 1–15 years were found [93]. Only two studies provided follow-up incentives for continuous PFMT during follow-up and long-term adherence to PFMT varied between 10% and 70%. For the POP studies found in the present review, there were no incentives for continuous PFMT after cessation of the original intervention, and only Barber et al. (2014) [62] reported long-term adherence to PFMT. They found that 93.4% reported to adhere to PFMT 6 months after the intervention and 81.4% at 2 years. However, there was no effect of PFMT in this study on either PFM strength or POP. In the 5-year follow-up of the Barber et al. study (2014) [62], Jelovsek et al. (2018) [67] reported that the estimated surgical failure rate was 61.5% and 70.3% in the two surgical groups, respectively. They concluded that rates of surgical failure increased during the follow-up period, although prolapse symptom scores remained improved.

There are challenges in adherence to long-term PFMT when there is no structured follow-up regimen or encouragement for further training after the initial supervised PFMT. This could explain the diminishing effect. Furthermore, if there was no short-term effect of a PFMT program [49, 50, 62, 67, 71, 75], then long-term effects are unlikely. Long-term success of any exercise training program relies on maintenance training. When women are left alone to do PFMT with no further incentives this is a challenge to some. The promotion of PFMT from a women’s health perspective should include strategies to support building PFMT into their general exercise/physical activity programs and/or daily routines. However, today < 30% of the adult population follows the WHO’s recommendations for participation in physical activity and exercise training, and it is likely that only those who are already physically fit and motivated for general training will attend structured exercise classes which could include specific PFMT [95–97]. New ways of reaching women with information on why and how to perform PFMT in addition to more general physical activity are needed. Due to injuries and weaknesses of the PFM and inability to contract correctly, PFMT may be even more difficult to adhere to than other exercise. More work is required to improve women’s long-term adherence to pelvic floor rehabilitation programs.

At present, one can question whether today’s health services for non-surgical interventions for POP are optimal. In a focus group study of 22 women receiving prolapse care through urogynecology services in the UK [98], the authors found that women often delayed seeking help for their symptoms because of lack of awareness, embarrassment and stigma. When they did present to their family doctors, their symptoms were often dismissed and unaddressed until they became more severe, and the women reported receiving little or no choice in treatment decisions. Choices were often influenced by health professionals' preferences and inconsistent with women's preferences and needs. Physical therapy-based interventions were reported as helping women regain control over their symptoms and life.

The results of the present narrative review support the conclusion of the ICI 2017 with 1A evidence/recommendation for PFMT in treatment of POP in the general female population [26]. In addition, the NICE guidelines (2019) [27] recommend considering a program of supervised PFMT for at least 16 weeks as a first option for women with symptomatic POP-Q stage I or stage II. If the program is beneficial, they recommend advising women to continue PFMT. There is no strong evidence for strength training maintenance programs, but intensity of the contraction seems to be the main factor. We therefore recommend 1–2 sessions a week with 8–12 close to maximum contractions. The overall best results found in our review were in an RCT with 18 supervised training sessions over a 6-month training period [25, 43]. We were only able to find one study on predictors of success of PFMT in women with POP [99]. They found that young age and having ≥ 1 indicators of obstetric trauma were predicting factors but discussed that many factors such as PFM function were not included in the analysis. We agree with the authors that there is a need for further studies on this important clinical question.

#### Clinical recommendations for effective PFMT for POP

*Provide proper information about the pelvic floor, exercise physiology and evidence for how PFMT can improve pelvic floor morphology and POP

*Teach proper PFM contraction technique and assess whether the woman can do a correct contraction (vaginal palpation, manometry, dynamometry, ultrasound) and give immediate feedback of performance

*Use validated outcome measures to assess POP symptoms, stage of POP (POP-Q) and comorbidities before and after treatment

*Offer individual weekly visits or group training with a physical therapist for at least 6 months with additional home training

*Teach women at risk for POP strategies to reduce IAP during activities of daily living

*Use motivational strategies to favor adherence

*Strength training regimens should follow general principles for strength training (3 sets of 8-12 close to maximum contractions at least 3-4 times/week and continue for at least 4 months)

*Register adherence to both supervised PFMT and home training

*Assess PFM variables (resting value, PFM strength and endurance) before and after treatment

*Recommend a suitable PFMT maintenance program with intensity of the contraction being the most important factor (8-12 contractions 1-2 times/week)

#### Recommendations for further studies

*Primary and secondary prevention studies in the general female population and studies in women with POP within 12 months after childbirth

*Monitoring long-term adherence and study predictors of success of PFMT for POP including pelvic floor biological/anatomical factors
